# Expression of transport proteins in the rete mirabile of european silver and yellow eel

**DOI:** 10.1186/s12864-021-08180-2

**Published:** 2021-12-02

**Authors:** Gabriel Schneebauer, Victoria Drechsel, Ron Dirks, Klaus Faserl, Bettina Sarg, Bernd Pelster

**Affiliations:** 1 Institute of Zoology, University of Innsbruck, Innsbruck, Austria; 2 Center for Molecular Biosciences, University Innsbruck, Innsbruck, Austria; 3 Future Genomics Technologies, Leiden, The Netherlands; 4 Institute of Medical Biochemistry, Protein Core Facility, Medical University Innsbruck, Innsbruck, Austria; 5 Institut für Zoologie Leopold-Franzens-Universität Innsbruck, Technikerstr. 25, A-6020 Innsbruck, Austria

## Abstract

**Background:**

In physoclist fishes filling of the swimbladder requires acid secretion of gas gland cells to switch on the Root effect and subsequent countercurrent concentration of the initial gas partial pressure increase by back-diffusion of gas molecules in the rete mirabile. It is generally assumed that the rete mirabile functions as a passive exchanger, but a detailed analysis of lactate and water movements in the rete mirabile of the eel revealed that lactate is diffusing back in the rete. In the present study we therefore test the hypothesis that expression of transport proteins in rete capillaries allows for back-diffusion of ions and metabolites, which would support the countercurrent concentrating capacity of the rete mirabile. It is also assumed that in silver eels, the migratory stage of the eel, the expression of transport proteins would be enhanced.

**Results:**

Analysis of the transcriptome and of the proteome of rete mirabile tissue of the European eel revealed the expression of a large number of membrane ion and metabolite transport proteins, including monocarboxylate and glucose transport proteins. In addition, ion channel proteins, Ca^2+^-ATPase, Na^+^/K^+^-ATPase and also F_1_F_0_-ATP synthase were detected. In contrast to our expectation in silver eels the expression of these transport proteins was not elevated as compared to yellow eels. A remarkable number of enzymes degrading reactive oxygen species (ROS) was detected in rete capillaries.

**Conclusions:**

Our results reveal the expression of a large number of transport proteins in rete capillaries, so that the back diffusion of ions and metabolites, in particular lactate, may significantly enhance the countercurrent concentrating ability of the rete. Metabolic pathways allowing for aerobic generation of ATP supporting secondary active transport mechanisms are established. Rete tissue appears to be equipped with a high ROS defense capacity, preventing damage of the tissue due to the high oxygen partial pressures generated in the countercurrent system.

**Supplementary Information:**

The online version contains supplementary material available at 10.1186/s12864-021-08180-2.

## Background

In physoclist fishes, i.e. in fish in which the embryonic connection of the swimbladder to the esophagus is lost during early development, the swimbladder is filled with gas molecules by diffusion from the blood and from swimbladder gas gland cells [1]. To generate the required high gas partial pressures to drive diffusion gas gland cells in the swimbladder epithelium secrete acid into the blood, switching on the Root effect [2–6]. The resulting initial increase in oxygen partial pressure, the so called single concentrating effect [7], is then in a second step multiplied by countercurrent concentration in the rete mirabile of the swimbladder [6–9]. The rete has been considered to function by passive diffusion and hydraulic (osmotic) transport [10–12], although Steen [13] suggested that lactate may be transported from the venous to the arterial side in the rete mirabile of the European eel. A re-assessment of lactate and water movements in the rete by measurement of hemoglobin and of metabolite concentrations in blood samples collected anterior and posterior to the rete mirabile of the European eel revealed a significant back-diffusion of lactate from venous capillaries to the arterial side, but no significant osmotic gradient and no water movement was detected [14]. This would require presence of lactate transport proteins in the rete, i.e. presence of monocarboxylate carrier proteins [15]. Including the back-diffusion of solutes in the rete and the salting-out effect, i.e. the reduction of gas solubility with increasing solute concentration in blood [16], in model calculations revealed that the back-diffusion of solutes even enhances the countercurrent concentrating capacity of a rete [17]. The possible presence of transport proteins in rete mirabile membranes, indicated by the recorded lactate movements in the rete, therefore could significantly support the countercurrent concentrating ability of the rete and thus enhance the capacity of the rete to generate elevated gas partial pressures. This would imply that the rete mirabile is not just a passive exchanger, and the countercurrent concentrating ability of the rete could not only be modified by changing the surface area of the capillaries, but also by modifying the expression of transport proteins in rete capillaries.

In many fish, the arterial capillaries of the rete mirabile are in intimate contact with the gas gland cells and in close proximity to the venous capillaries of the rete mirabile [18, 19]. Thus, collection of arterial and venous blood samples at the swimbladder side of the rete is difficult without contamination by the secretory activity of gas gland cells. In Anguillidae, such as the European eel, however, the two retia mirabilia of the swimbladder are bipolar and clearly separated from the gas gland cells. Therefore, blood samples can be collected at the arterial entrance and exit of the rete, and at the venous entrance and exit of the rete. For this reason, the eel has become a model species for the analysis of swimbladder physiology [13, 20, 21]. In the eel, each rete consists of about 30,000 to 40,000 arterial capillaries, which on the swimbladder pole of the retia give raise to two or three larger arterial vessels supplying the swimbladder epithelium, consisting of gas gland cells. From there the venous blood returns in two or three larger veins to the retia mirabilia, forming 20,000 to 30,000 venous capillaries, running parallel to the arterial capillaries with a diffusion distance of about 2 μm [22, 23].

The European eel is a catadromous fish spending most of its life cycle as so-called yellow eel in the European freshwater system. In preparation of the spawning migration eels pass the process of silvering to prepare for the transition to seawater. Silver eels then return to the spawning grounds in the Sargasso Sea, a journey taking about 5 to 6 months, perhaps even longer [24]. During silvering, which has been described as a secondary metamorphosis and puberty like event [25, 26], the size of the eel rete mirabile increases, indicating an improvement of the countercurrent concentrating ability [6, 7]. For American eel a two- to three-fold increase in rete length has been reported, and in the Japanese eel a 1.6-fold increase has been detected [27, 28]. Recent tracking studies revealed that migrating silver eels perform daily vertical migrations covering depth changes of several hundred meters [24, 29, 30]. The concomitant changes in hydrostatic pressure significantly affect the volume of the flexible-walled swimbladder, and it has been assumed that swimbladder function is improved during the process of silvering [24, 31]. Previous studies revealed significant changes in the transcriptome and the proteome of swimbladder gas gland cells associated with silvering, although the swimbladder is not involved in any osmoregulatory phenomenon. We therefore hypothesized that the countercurrent concentrating capacity of the rete mirabile, the second essential component of a physoclist swimbladder [1], would also be improved during silvering. We hypothesized that the countercurrent concentrating capacity of the rete mirabile would be supported by the expression of solute transport proteins in rete capillaries. Furthermore, during silvering the expression of these proteins would be enhanced, providing further support for the countercurrent concentration. To test these hypotheses, we analyzed and compared the transcriptome as well as the proteome of rete mirabile tissue of European yellow and silver eels. Our previous studies on gas gland cells could only be performed on separate tissue samples, so that a correlation between transcriptome and proteome could not be tested. We therefore aimed at obtaining both, transcriptome and proteome data from each individual fish, in order to connect transcriptome and proteome data.

## Results

On average (5 yellow eels; 6 silver eels), a cDNA library was sequenced at a depth of ~ 16 mio raw reads. Alignment to the European eel reference genome [32] resulted in about 71% mapped gene reads and 32,674 transcripts that could be hit by at least one read in the transcriptome. Comparing the transcriptome of yellow and silver eels, 99 differentially expressed genes were detected at the level of p < 0.01, 79 of these genes could be assigned to a known function. Restricting this analysis to genes expressed with a relative expression value higher than 50 (base mean value) resulted in only 47 genes that were differentially transcribed, out of which 41 had a predicted function (Table 1). Therefore, the data sets were combined for further analysis to identify genes that are generally transcribed in rete endothelial cells of European yellow and silver eels.

**Fig. 1:**
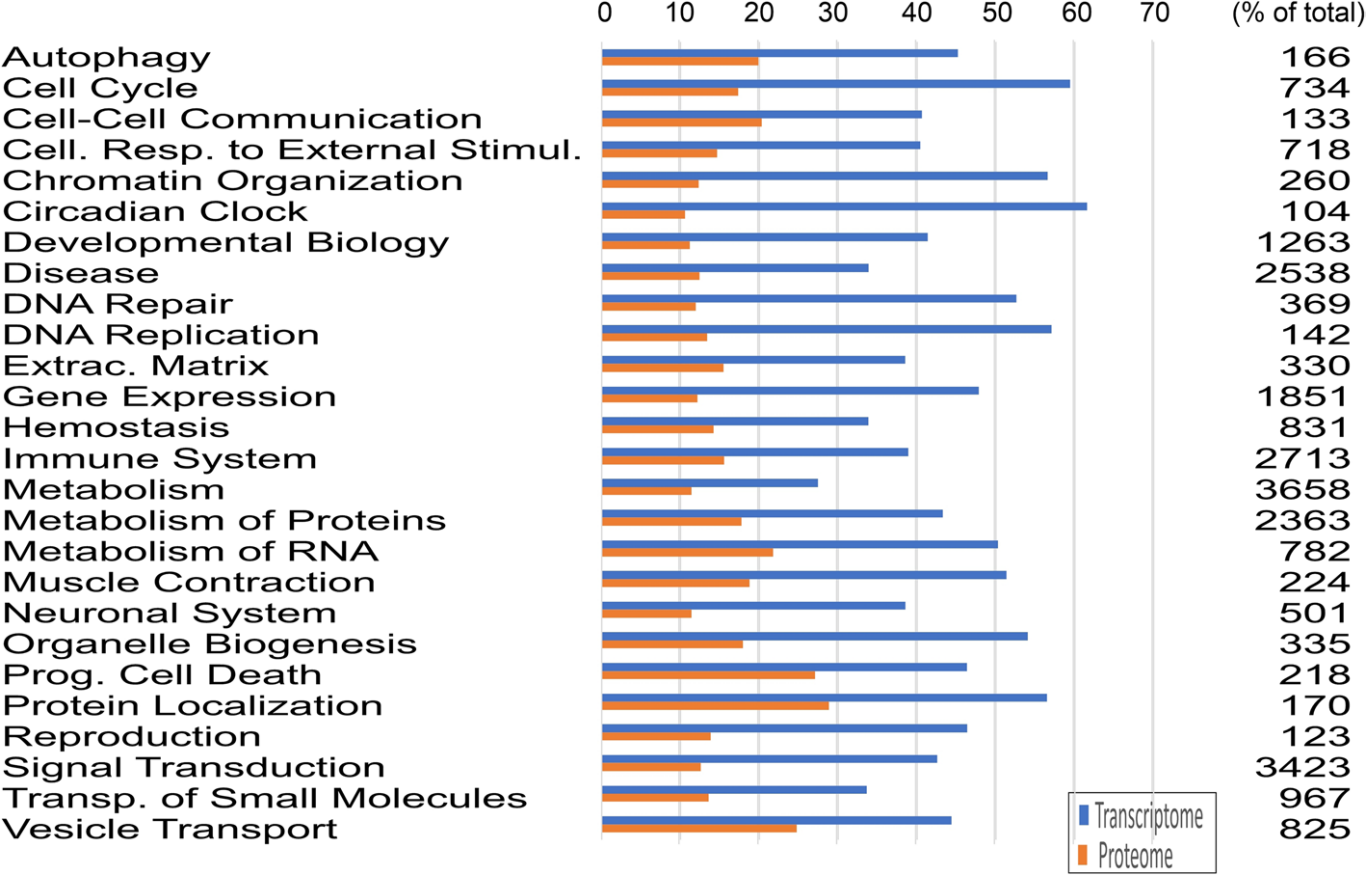
Percentage of genes of the 25 pathways identified in the Reactome analysis in the transcriptome (blue line) and the proteome (orange line) of European yellow and silver eel rete mirabile tissue. Because only very few genes were differentially expressed between yellow and silver data sets were combined to obtain a clearer picture of genes expressed in rete mirabile tissue. The number gives the total number of genes listed in the Reactome data base for the respective pathway

Pathway analysis using the Reactome pathway browser revealed that in 25 pathways between 27% and 61% of the in the Reactome database listed genes were transcribed in rete tissue of the European eel (Fig. 1). 59% of cell cycle related genes were transcribed, but also 34% of genes involved in transport of small molecules and 44% of genes connected to vesicle transport. Of note, 43% of the genes involved in signal transduction were transcribed in rete tissue. Of the genes associated with metabolism, 49 out of 140 genes were involved in glucose metabolism and 14 out of 46 genes associated with the pentose phosphate shunt were detected. In addition, 60 out of 233 genes involved in the citric acid cycle and 39 out of 150 genes contributing to the respiratory chain were transcribed in rete tissue. Looking at genes responsible for the formation of the extracellular matrix, 127 out of 330 listed genes were detected in the rete transcriptome.

**Table 1:** Between yellow and silver eels differentially expressed genes detected in the transcriptome with a base mean value > 50. (p < 0.01). Using a more stringent p-adjust value none of these genes is significantly different

Name	Description	baseMean	pval
*abcg2*	atp-binding cassette sub-family g member 2	55	5,38E-04
*acc2a*	amiloride-sensitive cation channel 2- neuronal	67	1,66E-05
*angl4*	angiopoietin-related protein 4	1302	8,71E-03
*ats17*	a disintegrin and metalloproteinase with thrombospondin motifs 17	493	2,38E-03
*axn2*	axin-2	50	3,78E-03
*c85cb*	coiled-coil domain-containing protein 85c-b	105	9,25E-03
*ceam5*	carcinoembryonic antigen-related cell adhesion molecule 5	144	6,96E-03
*cooa1*	collagen alpha-1 chain flags: precursor	126	3,50E-03
*edil3*	egf-like repeat and discoidin i-like domain-containing protein 3	133	2,41E-03
*edn2*	endothelin-2	430	4,53E-03
*fras1*	extracellular matrix protein fras1 flags: precursor	787	1,92E-06
*gbgt1*	globoside alpha- -n-acetylgalactosaminyltransferase 1	69	9,94E-03
*gpda*	glycerol-3-phosphate dehydrogenase cytoplasmic	356	5,16E-04
*gpda*	glycerol-3-phosphate dehydrogenase cytoplasmic	226	6,40E-03
*gtd2b*	general transcription factor ii-i repeat domain-containing protein 2b	73	4,60E-03
*ha18*	h-2 class i histocompatibility q8 alpha chain flags: precursor	63	7,96E-03
*hebp2*	heme-binding protein 2	3544	1,17E-03
*hrsl1*	hras-like suppressor	534	9,16E-03
*hs3s1*	heparan sulfate glucosamine 3-o-sulfotransferase 1	558	2,87E-03
*ic1*	plasma protease c1 inhibitor	905	4,32E-03
*kalm*	anosmin-1	129	4,77E-05
*lgi1*	leucine-rich glioma-inactivated protein 1 flags: precursor	160	9,80E-03
*lyg*	lysozyme g	192	6,21E-03
*mboa2*	membrane-bound o-acyltransferase domain-containing protein 2	56	6,91E-03
*mica2*	protein mical-2	65	7,23E-03
*mmp11*	stromelysin-3	91	6,21E-03
*mrp*	marcks-related protein	2803	8,04E-03
*nptx1*	neuronal pentraxin-1	116	2,02E-03
*nrx3a*	neurexin-3-alpha	82	4,00E-04
*ntng1*	netrin-g1	73	6,48E-03
*pkha6*	pleckstrin homology domain-containing family a member 6	152	9,60E-03
*pkp1*	plakophilin-1	78	8,14E-03
*rrmj3*	rrna methyltransferase 3	1440	3,47E-03
*rrmj3*	rrna methyltransferase 3	141	7,01E-03
*s4a7*	sodium bicarbonate cotransporter 3	133	3,05E-05
*scm2b*	calcium-binding mitochondrial carrier protein s -2-b	133	6,58E-03
*scrb2*	lysosome membrane protein 2	3758	3,52E-05
*smox*	spermine oxidase	236	8,16E-03
*sp5*	transcription factor sp5	120	9,40E-03
*visl1*	visinin-like protein 1	54	7,96E-03
*wn10a*	protein wnt-10a flags: precursor	127	3,63E-03

A more detailed analysis of the transcriptome was restricted to genes transcribed with a relative expression value higher than 50. Among the transcripts related to the formation of the extracellular matrix, 15 collagen transcripts were detected (*cola, coaa, co6a, coca, co4a, co5a, coga, co2a, cra1b*), and also one collagenase transcript (Additional file 1). Additional transcripts of extracellular matrix constituents were 6 laminins (*lamc, lama*), and basement membrane specific proteoglycan transcripts (*pgm*) were detected. The transcriptome also contained transcripts of matrix metalloproteinases and of collagenase (*mmp*, *ats*).

**Fig. 2:**
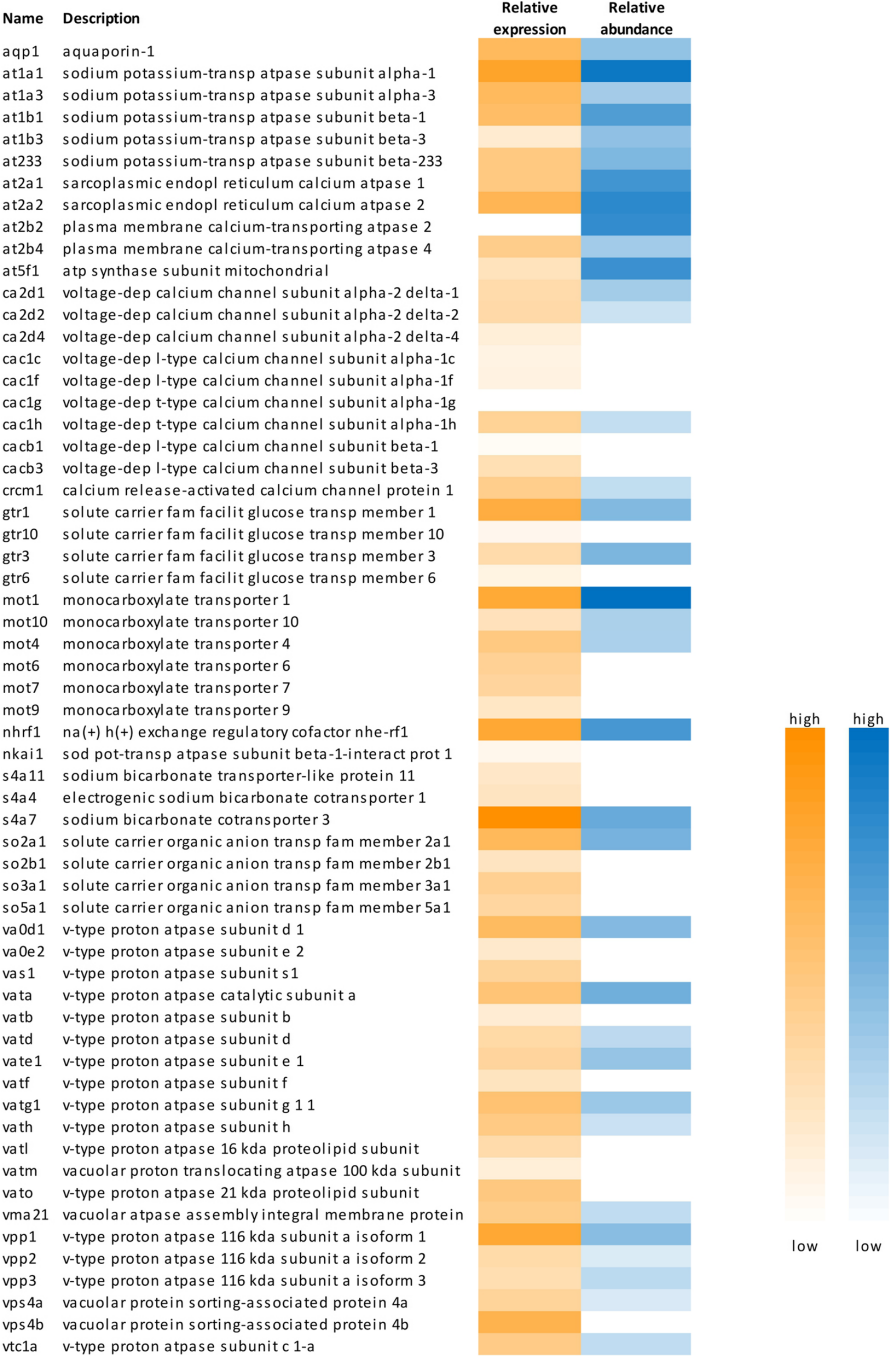
Heat map showing selected membrane transport proteins detected in the transcriptome and/or in the proteome of European yellow and silver eel rete mirabile tissue samples. Relative expression values (yellow) range from 50 to 4000, relative protein abundance (blue) from 10^6^ to 3.5*10^9^

Searching the transcriptome with respect to transcripts translating into membrane transport proteins (ion transport proteins; channel proteins; membrane ATPases) (Fig. 2; Additional file 2) revealed the presence of 33 transcripts of V-type proton ATPase subunits, coding for 15 different subunits (*vata, vatb, vatd, vate, vatf, vatg, vath, vatl, vatm, vato, vpp, va0d, va0e, vtc1*). The family of calcium channel proteins was also represented by a large number of transcripts (*ca2d, cac1h, cac1, cacb, crcm*).

Several transcripts of various members of the solute carrier families (SLC) were detected, including transcripts of sodium bicarbonate transport proteins (*s4a*) and transcripts of different SLC families involved in organic anion transport (*so2, so3, so5)*. 6 different monocarboxylate transporter transcripts (*mot1*, *mot4, mot6, mot7, mot9, mot10*) were detected, and a number of different glucose carrier transcripts were expressed (*gtr1, gtr3, gtr6, gtr10*) (Fig. 2; Additional file 2).

Focusing in particular on signal transduction and receptor related transcripts revealed the presence of receptor type tyrosine-protein phosphatase (*ptpr*) and of vascular endothelial growth factor receptor (*vgfr*) transcripts. In addition, endothelin receptor transcripts (*ednra, ednrb*) acetylcholine receptor (*acha*) and adrenergic receptor (*ada, arbk*) were found. Transforming growth factor beta receptor beta (*tgbr, tgfa, tgfr*) and inositol-trisphosphate receptor (*itpr*) were also detected in the transcriptome (Additional file 3).

Analysis of the proteome revealed 5289 proteins, out of which only 104 proteins were uncharacterized proteins with yet unknown function (Additional file 4). In the proteome of yellow and silver eels, 74 proteins were differentially expressed, 73 of these proteins had a predicted function (Additional file 5). 67 proteins were reduced in their expression level, but only 2 proteins (synembryn-A and fucolectin-7like) were reduced by more than 3-fold. 7 proteins were about 2-fold elevated in their expression level. Accordingly, the changes in protein abundance between silver and yellow eels were modest. Noteworthy, among the proteins significantly affected were mimecan and several collagens, components of the extracellular matrix (Additional file 5), but no membrane transport proteins.

**Fig. 3:**
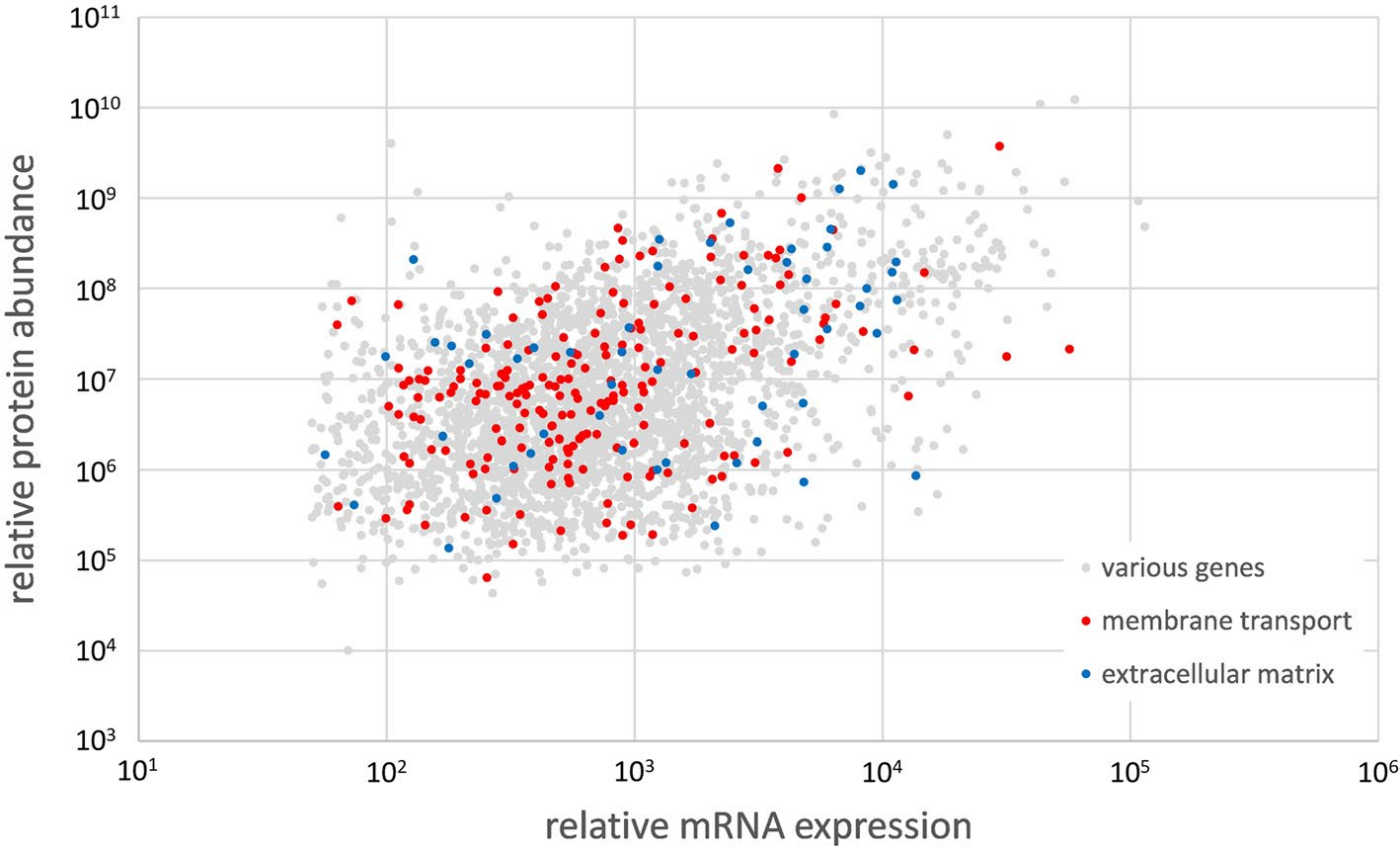
Relative mRNA expression plotted versus the relative protein abundance of all genes detected in the proteome as well as in the transcriptome of European yellow and silver eel rete mirabile tissue samples. Genes associated with membrane transport are marked in red, genes associated with the extracellular matrix are marked in blue. Spearman’s rank-order correlation: rs(2851) = 0.422, p = 0.000000200

For a more detailed data analysis of proteins that are expressed in rete capillaries, the procedure developed for the transcriptome was adopted and the data sets of yellow and silver eels were combined. Pathway analysis using the Reactome pathway browser revealed that, on the basis of 5185 proteins with known function, in 25 pathways between 10% and 27% of the Reactome database listed genes were translated in rete tissue of the European eel (Fig. 1). Overall, 36% of the genes detected in the transcriptome were also detected in the proteome. The lowest match with only 17% was detected for circadian clock genes, the highest agreement was present for genes related to apoptosis with 58%. Table 2 shows the overlap of proteome and transcriptome data for the specific physiological functions addressed in this analysis. The highest overlap was detected for ATPases. For 84% of the detected transcripts the appropriate proteins were found. On the other hand, only 41% of the transcripts coding for receptor proteins the appropriate proteins could be detected. In Fig. 3 for all genes detected in both, the transcriptome as well as in the proteome, relative mRNA expression values are plotted versus the relative protein abundance. Relative mRNA expression of most genes was in the range 10^2^ to 10^4^, while protein abundance varied between 10^5^ and 10^8^. Spearman’s rank-order correlation was run to determine the relationship between mRNA expression and protein abundance. There was a moderate, positive correlation between mRNA expression and protein abundance, which was statistically significant (rs(2851) = 0.422, p = 0.000000200).

**Table 2:** Number of genes in specific functional groups detected in the transcriptome and in the proteome (Trans. + Prot.), and the number of genes detected either in the transcriptome or in the proteome (Trans. or Prot.). The fractional overlap between transcriptome and proteome is given as % value

	**Trans. + Prot.**	**Trans. or Prot.**	**%**
Channel	54	41	57
ATPase	175	34	84
Extr. matrix	130	176	42
Metabolism	2147	1404	60
Mitochondria	946	336	74
Ion transport	523	351	60
Receptor	257	369	41

**Fig. 4:**
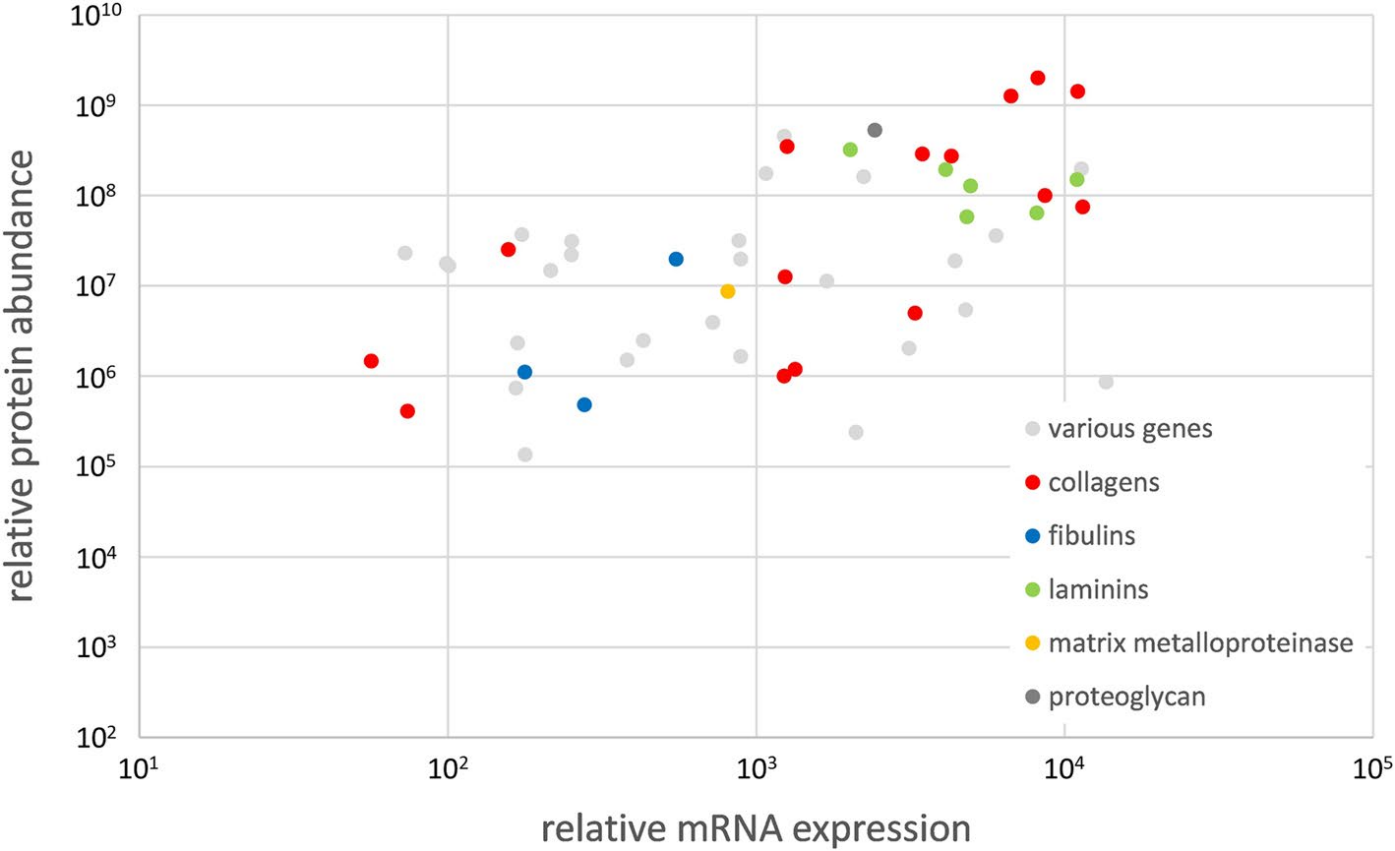
Relative mRNA expression plotted versus the relative protein abundance of selected extracellular matrix genes detected in the proteome as well as in the transcriptome of European yellow and silver eel rete mirabile tissue samples. Different families of extracellular matrix proteins are color coded

Searching the description of the annotated proteins for terms related to the extracellular matrix a large number of proteins were detected (Additional file 1). Consistent with the transcriptome, these proteins included a number of collagens (Co6a, Coca, Coea, Co18a, Col18a, Cola1) and several laminins (Lama, Lamc). In contrast to the transcriptome, disintegrin and metalloproteinase (Ats) were not detected in the proteome. In Fig. 4, the relative mRNA expression of the extracellular matrix protein families is plotted versus the relative protein abundance. In addition to laminins and the collagen protein family, fibulins and a proteoglycan were detected, and all families were present with similar relative expression values and protein abundances.

**Fig. 5:**
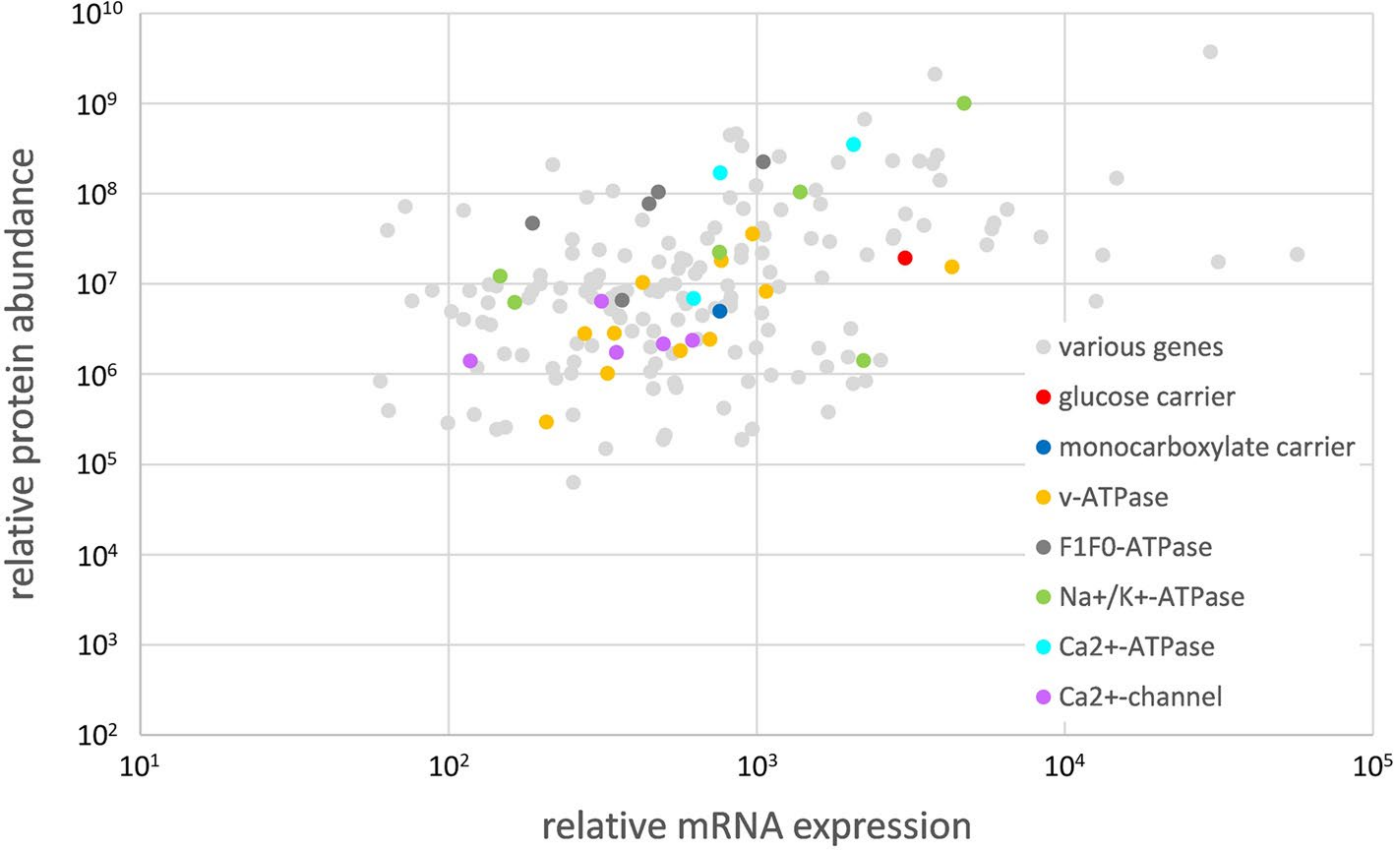
Relative mRNA expression plotted versus the relative protein abundance of selected genes coding for proteins involved in membrane transport detected in the proteome as well as in the transcriptome of European yellow and silver eel rete mirabile tissue samples

Focusing on membrane transport (Fig. 2; Additional file 2), proteins contributing to 13 subunits of V-type proton ATPase were isolated (Vaod, Vata, Vatd, Vate, Vatf, Vatg, Vath, Vma21, Vpp, Vps, Vtc). Both subunits of Na^+^/K^+^-ATPase were detected (At1a1, At1a3; At1b1, At1b3, At233), and also subunits of plasma membrane Ca^2+^-ATPase (At2b2, At2b3, At2b4, Atp2c1) and sarcoplasmic reticulum Ca^2+^-ATPase (At2a1, At2a2) (Additional file 2). In the family of solute carrier proteins, the proteome included two glucose transport proteins (Gtr1, Gtr3), as well as several monocarboxylate carriers (Mot1, Mot4, Mot10). The heat map illustrates that genes expressed with high mRNA copy numbers typically were also highly expressed at the protein level, like, for example Gtr1 or Mot1. This was also observed for the highly expressed sodium bicarbonate cotransporter (S4a7) and a number of subunits of various calcium channel proteins (Ca2d1, Ca2d2, Cac1h), also present in the transcriptome at intermediate levels. Other calcium channel subunits present with a relatively low expression value in the transcriptome were not detected in the proteome (Fig. 2). Plotting the relative mRNA expression versus the protein abundance revealed the presence of a large number of membrane transport proteins and of a remarkable number of ATP-dependent transport proteins including the presence of most subunits of mitochondrial F_1_/F_0_-ATP synthase (Fig. 5). Most of these genes were expressed with a relative mRNA expression of 10^2^ to 10^4^ and a protein abundance of 10^6^ to 10^8^.

**Fig. 6:**
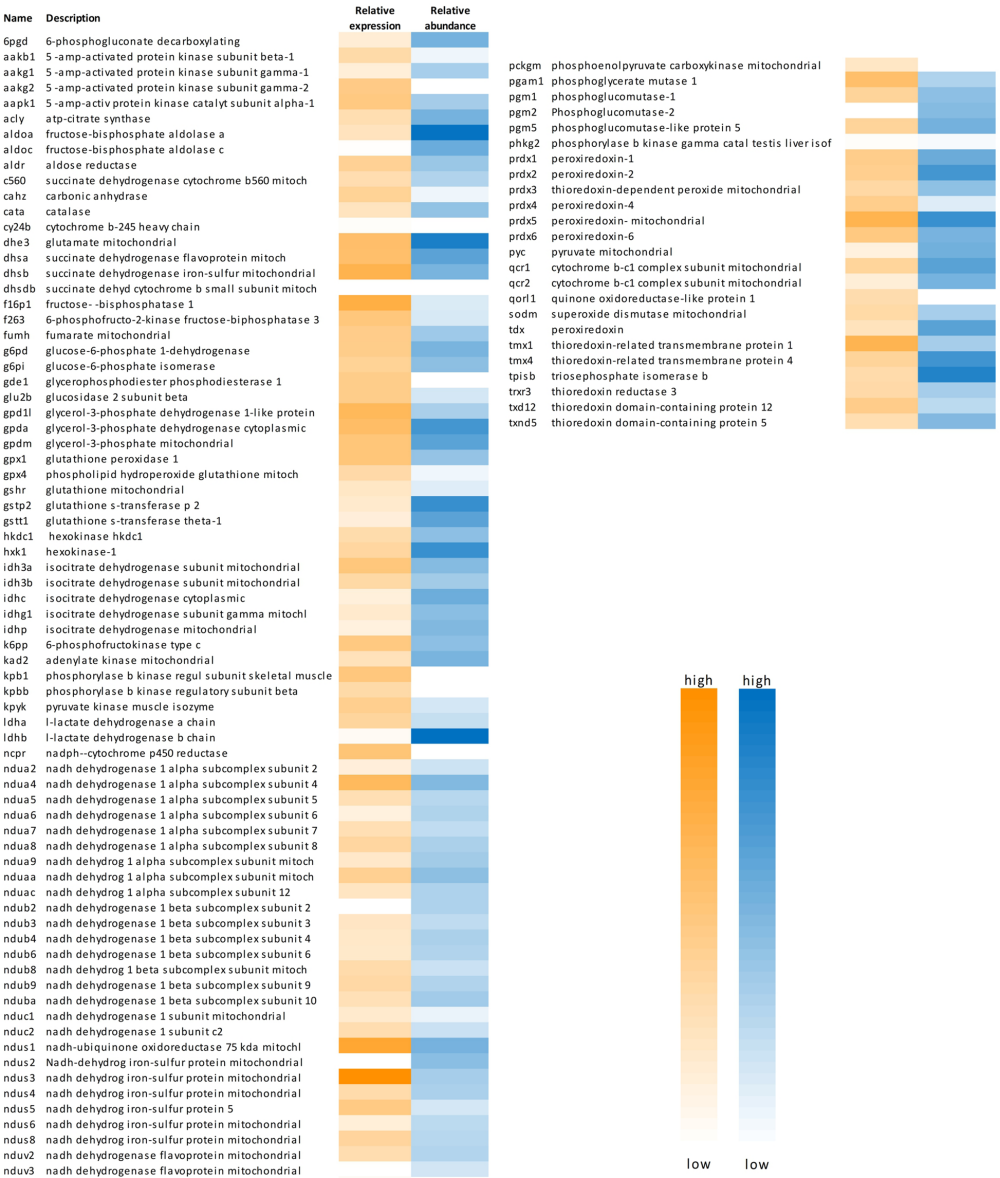
Heat map showing selected proteins involved in cellular energy metabolism detected in the transcriptome and/or in the proteome of European yellow and silver eel rete mirabile tissue samples. Relative expression values (yellow) range from 50 to 30,000, relative protein abundance (blue) from 10^6^ to 1.2*10^10^

A large number of proteins involved in glucose metabolism were identified (including Hkdc1, Hxk1, Aldr, K6pp, F16p1, F263, Gpda, Ldha, Ldhb, Kpbb), but also two proteins involved in the pentose phosphate pathway (6Pgd, G6pd). Proteins involved in mitochondrial energy metabolism included, for example, Acly, Dhsa, Dhsb, Idhc, Idh3, Ndua, Ndub, Nduc, and Ndus (Fig. 6). Overall, 459 mitochondrial proteins were detected in the proteome, including 39 proteins contributing to cytochrome complex formation. 139 of the detected mitochondrial proteins were involved in the biological process ‘electron transport’.

Noteworthy was the presence of proteins connected to the degradation of reactive oxygen species, including catalase (Cata), glutathione related proteins (Gshr, Gstp, Gstt), superoxide dismutase (Sodm), peroxiredoxin (Prdx, Tdx), and thioredoxin (Txd, Txnd).

In line with the transcriptome, the proteome included a number of receptor-type tyrosine-protein phosphatases (PTPR). In addition, receptor-tyrosine kinases (Tie1, Tie2, Tyro3), endothelin receptor (Ednra, Ednrb), beta adrenergic receptor kinase (Aarbk2) and atrial natriuretic peptide receptor (Anpra) were expressed at the protein level. Glucocorticoid receptor (Gcr) and insulin receptor short (Insr) were also found (Additional file 3).

## Discussion

Analysis of the transcriptome and of the proteome revealed presence of a large number of proteins of the families of SLC membrane transport proteins and of membrane bound ATPases, which was in line with our hypothesis that the countercurrent concentrating capacity of the rete mirabile would be supported by the expression of solute transport proteins in rete mirabile capillaries. Our hypothesis that during silvering the expression of these proteins would be enhanced, however, was not supported by the data.

### General comparison of transcriptome and proteome data

Overall comparison of the transcriptome and the proteome revealed that the number of proteins isolated in the proteome amounted to about 16% of the identified transcripts, and most of the detected proteins had a predicted function. In fish, the large number of transcripts certainly is related to the whole genome duplications encountered during evolution [33, 34]. Analysis of the proteome of European eel gas gland cells resulted in the identification of 6223 proteins [35], a value about 20% higher than the number of proteins isolated from rete cells. While in gas gland cells, all fractions have been analyzed [35], our rete cell analysis was focused on the membrane fraction, so that cytosolic proteins probably were underrepresented, which may have contributed to the lower number of proteins identified in the rete.

In contrast to previous studies on swimbladder gas gland cells, in which transcriptome and proteome data have been collected from different tissue samples [35–37], in our present study both data sets have been collected from the same tissue samples, allowing for an analysis of the correlation between protein and mRNA expression. The results revealed that in rete tissue, the overall correlation between protein and mRNA was modest with a Spearman’s correlation of 0.422, (p = 0.000000200).

Focusing on the overlap between transcriptome and proteome within functional groups, the results varied remarkably. While for mitochondrial proteins and ATPases an overlap of 74% and 84% was detected, respectively, less than 50% of the transcripts coding for receptor proteins were detected in the proteome, and overall, the overlap was even lower with a value of 36%. While in yeast cells, a good correlation between detected protein copies and mRNA copies has been reported [38], other studies indicated a poor correlation between mRNA and protein levels in different tissues [39–41].

### The effect of silvering

In the eel, silvering is accompanied by an elongation of the rete mirabile, which improves the countercurrent concentration capacity [27, 28]. Silver eels, during their spawning migration, experience significantly larger hydrostatic pressures than yellow eels, dwelling in freshwater rivers and lakes. It therefore was expected that the elevated gas partial pressures necessary to fill the swimbladder at high hydrostatic pressure would require an increased countercurrent concentrating capacity of the rete mirabile and thus would affect the expression of membrane transport proteins. In previous studies it has already been demonstrated that silvering is associated with significant changes in the transcriptome and the proteome of swimbladder gas gland cells [35–37]. This obviously was not the case in the rete mirabile tissue as only about 0.2% of the transcripts were significantly different in silver eels at the level of p < 0.01, and using p-adjust for the analysis none of these genes was significantly different. Analysis of the gas gland cell transcriptome of yellow and silver eels revealed 646 differentially expressed genes at the level of p < 0.01 [36].

Our proteome analysis confirmed the analysis of the transcriptome. Less than 1% of the proteins were significantly affected in the rete of silver eels, the change in abundance was moderate, with only two genes reduced more than 3-fold in their relative abundance. None of the membrane transport proteins was affected. Our data thus did not reveal large differences in rete gene expression between yellow and silver eels. Our tissue samples were collected from yellow eels and from silver eels after completing the transition, not during silvering. In silver eels, the length of the rete capillaries is increased [27, 28], resulting in a larger overall surface area available for back-diffusion and countercurrent concentration. Our data thus suggest that in contrast to our expectation during silvering hardly any changes in the area specific transport capacity are encountered. Increasing length and thus surface area of the rete capillaries [27, 28] increases tissue mass, but this apparently was not associated with a modulation of gene expression. Increasing the surface area of the rete appears to be the main effect allowing for an enhanced countercurrent concentration ability in silver eels.

### Membrane transport proteins

We hypothesized that in the rete the countercurrent concentrating capacity would be supported by the expression of specific membrane transport proteins facilitating back-diffusion of small molecules (ions and metabolites) from the venous to the arterial side. The presence of a large number of ion transporter and of monocarboxylate transport proteins indeed suggested that rete capillaries are not only passive exchangers, but transport of ions and metabolites can be supported by specific transport proteins. Presence of monocarboxylate transport (Mot) proteins (=MCT proteins; SLC16) was in line with the previously detected back-diffusion of lactate from venous to arterial rete capillaries [14]. *Mct1* and *mct4* mRNA has also been detected in arterial capillaries of the rete mirabile and in gas gland cells of fugu (*Takifugu rubripes*) by in situ hybridization [42], suggesting that the expression of transport proteins is a widespread phenomenon in swimbladder rete mirabile tissue. Back-diffusion of lactate has been shown to be advantageous for the salting out effect [17], and this is particularly important for the concentration of inert gases. The transport proteins identified in rete endothelial cells therefore may contribute to back-diffusion of solutes and elevate the inert gas partial pressure, which would allow for countercurrent concentration of inert gases [16]. Numerous subunits of a V-type proton ATPase have been detected in the transcriptome as well as in the proteome, and a V-type proton ATPase may contribute to back-diffusion of protons from the venous to the arterial side of the rete and support the acidification of arterial rete capillaries. Back-diffusion and countercurrent concentration of acidic metabolites has been shown to enhance blood acidification, switching on the Root effect [6, 21].

Remarkable was the presence of a large number of calcium channel protein transcripts and proteins. In addition, various subunits of cytoplasmic calcium ATPase as well as of endoplasmic reticulum calcium ATPase (SERCA) were identified, indicating extensive Ca^2+^ movements between the endoplasmic reticulum and the cytoplasm, and also between the extracellular space and the cytoplasm. These movements probably are of regulatory importance, perhaps influencing the tightness of the connection between endothelial cells. This would be in line with the presence of a large number of receptor tyrosine kinase and phosphatases, including the angiopoietin receptors TIE1 and TIE2. Angiopoietin is a crucial growth hormone for angiogenesis, which is required for elongation of rete capillaries, as observed during silvering in eels. Many receptor tyrosine kinases are receptors for growth hormones, and reversible receptor tyrosine kinase phosphorylation is a very important mechanism contributing to cell adhesion, cell proliferation and cell-cell interaction [43]. Ca^2+^ movements could also contribute to the control of blood flow through the rete, and thus the swimbladder tissue. Noteworthy in this context was the detection of endothelin receptor, beta-adrenergic receptor kinase and of the ANP receptor. The activity of smooth muscle cells at the entrance of the rete could perhaps modify perfusion resistance and thus control blood flow through the swimbladder. The secretory activity of swimbladder tissue has been shown to be dependent on blood flow through the swimbladder [44].

### Energy metabolism

Transport of ions or solutes in a second step often involves the activity of Na^+^/K^+^-ATPase, and the subunits of this ATPase have been detected in the transcriptome as well as in the proteome. The ATP-consumption of these ATPases can be covered by oxidative phosphorylation as indicated by the presence of F_1_F_0_-ATP synthase.

A large number of genes related to energy metabolism were detected in rete endothelial cells, including many enzymes of the glycolytic pathway. This was expected, as studies by Rasio [45, 46] suggested that the energy metabolism of rete endothelial cells is almost exclusively based on glycolysis and lactate production. Our results, however, also show the presence of the two key enzymes of the pentose phosphate shunt (PPS). The PPS results in the generation of NADPH_2_, which is required for the glutathione metabolism and thus involved in ROS degradation, which appears to be important for rete tissue (see below). In addition, our data reveal the presence of a large number of mitochondrial transcripts with high transcript numbers: 21 transcripts of various ATP synthase subunits and 41 cytochrome transcripts. Also, ADP/ATP translocase was among the mitochondrial transcripts with highest transcript values. This was in line with the detection of mitochondrial proteins including key enzymes of the citric acid cycle, NADH dehydrogenase and cytochrome oxidase from the respiratory chain, many subunits of F_1_F_0_-ATP synthase and ADP/ATP translocase. Aerobic ATP production is much more efficient than anaerobic ATP formation, and this appears to be related to the presence of various ATP dependent transport proteins, including Na^+^/K^+^-ATPase, V-type proton ATPase and Ca^2+^-ATPases located in cell membranes as well as in the endoplasmic reticulum. We therefore conclude that rete endothelial cell metabolism is not restricted to anaerobic ATP formation, but also includes aerobic ATP production in the oxidative metabolism.

### Extracellular matrix

Rete capillaries are surrounded by thin basement membranes [22, 47], and a large number of collagens and laminins have been detected in the transcriptome as well as in the proteome. Basement membranes of the European eel have been described as layered structures containing numerous microfibrils [22], and elongation of rete capillaries requires additional extracellular matrix proteins. Concomitant with basement membranes constituents, a number of matrix metalloproteases have been identified at the mRNA and at the protein level, which are required for maintenance and reconstruction of the basement membrane.

### Defense of reactive oxygen species

The swimbladder has been shown to contain high concentrations of oxygen, and with increasing depth increasing hyperoxic conditions prevail [48–50]. Analysis of the ROS defense capacity of swimbladder tissue of six marine fish suggested that a high oxygen content of the swimbladder is correlated with a high superoxide dismutase (SOD) activity [51]. A recent study on the European eel confirmed a high ROS defense capacity and showed that SOD activity in swimbladder tissue of the yellow eel was significantly higher than in muscle tissue [52]. During silvering, a process preparing the yellow eel for the spawning migration to the Sargasso Sea, SOD activity was even elevated. The high oxygen partial pressures required to fill the swimbladder are generated in the rete mirabile. Therefore it is not surprising that rete endothelial cells also are characterized by a high ROS defense capacity, as shown in the present study. Catalase, superoxide dismutase, glutathione related enzymes, peroxiredoxin and also thioredoxin were all detected at the mRNA as well as at the protein level, and the significant changes observed in the proteome of silver eels include slight elevations in reactive oxygen species degrading enzymes (SOD, peroxiredoxin, glutathione-S-transferase). These results confirm the conclusion that fish tissues exposed to high oxygen partial pressures require a high ROS defense capacity [53].

In summary, our data indicated that in swimbladder rete tissue a remarkable set of transport proteins was expressed, and in this respect the transcriptome and the proteome data were remarkably consistent. This suggested that protein mediated transport significantly supports back-diffusion of ions and metabolites in the rete mirabile, which enhances the countercurrent concentrating capacity of the rete. Silvering, however, was connected to only moderate changes in protein expression and did not affect the expression of membrane transport proteins. We therefore have no indication for an improvement of area specific transport capacities of the rete associated with silvering. The main effect of silvering on the countercurrent concentrating capacity of the rete appears to be achieved by increasing the total surface area. As already demonstrated for swimbladder tissue, the hyperbaric oxygen tensions generated in the rete mirabile coincide with a high ROS defense capacity in rete cells.

## Methods

Six yellow and six silver female eels were caught by commercial fishermen in Lake Constance, Bregenz, Austria, with bottom traps. They were transferred to the Institute of Zoology at the University of Innsbruck and kept in the freshwater aquarium until sampling the tissue. Table 3 summarizes the morphometrics of yellow and silver eels used for the experiments.

**Table 3:** Morphometrics of European eels presented as mean ± SD, silvering index according to Durif et al. (64), and ocular index according to Pankhurst (65)

	Yellow eels (n = 6)	Silver eels (n = 6)
Body mass [g]	392.7 ± 149.1	1037.5 ± 327.1
Body length [cm]	65.3 ± 7.5	86.5 ± 6.5
Pectoral fin length [mm]	27.6 ± 3.9	41.6 ± 2.6
Horizontal eye diameter [mm]	6.8 ± 0.8	10.5 ± 1.0
Vertical eye diameter [mm]	6.3 ± 0.6	10.2 ± 0.8
Silver Index	2.5 ± 0.5	4.5 ± 0.5
Ocular Index	5.2 ± 0.5	9.8 ± 1.1

### Preparation of rete tissue

Under anesthesia with 2-Phenoxyethanol (1 ml*L^− 1^) eels were decerebrated and spinally pithed. Eels were placed into an ‘eel holder’, as described by Pelster et al. [54]. The gills were irrigated with well-oxygenated tap water (flow rate 4-5 L*min^− 1^). The body wall was opened ventrally, the swimbladder was exposed and carefully freed from connective tissue. Artery and vein at the heart pole of the retia mirabilia were carefully separated and occlusively cannulated using PE50 and PE60 catheters, respectively. Rete vessels were perfused with heparinized (100 i.u*ml^− 1^) saline solution containing (in mmol*L^− 1^) NaCl, 129; KC1, 5; MgSO_4_, 0.9; CaCl_2_, 1.1; glucose, 15. Perfusion of rete vessels was controlled by continuous binocular inspection and both retia mirabilia were dissected immediately after total clearance of blood cells. Half of the tissue was blotted dry on absorbent paper, shock frozen in liquid nitrogen and stored at -80^o^C until further use for analysis of protein expression. The second half was transferred into 1 ml RNAlater™ solution (Invitrogen by Thermo Fisher Scientific Inc., Waltham, MA, USA) and immediately shock frozen in liquid nitrogen for analysis of the transcriptome.

### RNA isolation and Illumina RNAseq analysis

Total RNA of rete mirabile tissue of five yellow and six silver eels was isolated using the Qiagen miRNeasy Mini kit and a TissueRuptor according to the manufacturer’s instructions (Qiagen, Venlo, Netherlands). Quality and integrity of the isolated RNA were checked on an Agilent Bioanalyzer 2100 total RNA Nano series II chip (Agilent, Amstelveen, Netherlands). The Illumina Truseq library prep started with polyA+ RNA selection step, thereby selecting against rRNA. Illumina RNAseq libraries were prepared from 0.5 µg total RNA using the Illumina TruSeq Stranded mRNA Library Prep according to the manufacturer’s instructions (Illumina Inc. San Diego, CA, USA). All RNAseq libraries (150-750 bp inserts) were sequenced using an Illumina NovaSeq6000 system as 2 × 150 nucleotides paired-end reads or an Illumina HiSeq2500 system as 1 × 50 nucleotide single-reads according to the manufacturer’s protocol. Image analysis and base calling were done using the Illumina pipeline.

### Illumina data processing

Data processing was performed as described previously [55, 56]. Briefly, reads (16 million per sample) were aligned to the draft genome sequence of European eel [32] using TopHat (version 2.0.13) https://ccb.jhu.edu/software/tophat/index.shtml [57]. Secondary alignments of reads were excluded by filtering the files using SAMtools (version 1.2 using htslib 1.2.1) [58]. Aligned fragments per predicted gene (also referred to as transcripts) were counted from SAM alignment files using the Python package HTSeq (version 0.6.1p1) https://readthedocs.org/projects/htseq/ [59]. To enable comparisons across samples, fragment counts were corrected for the total amount of sequencing performed for each sample. As a correction scaling factor, library size estimates, determined using the R/Bioconductor (release 3.3.2) package DESeq https://bioconductor.riken.jp/packages/3.4/bioc/html/DESeq.html [60], were employed. Read counts were normalized by dividing the raw counts obtained from HTSeq https://htseq.readthedocs.io/en/master/install.html by its scale factor. The term “relative expression value” refers to these normalized read counts.

The Reactome database (https://reactome.org) [61] was used for classification of genes into broad pathways. For a detailed pathway and biological process analysis of expressed genes, analysis of the transcriptome was limited to transcripts with a base mean relative expression value above 50 copies in yellow and silver eel rete tissue. The *Description* of the transcript, GO *biological process* and *molecular function* were searched for genes related to a specific metabolic pathway or transport activity (like, for example, metabolic process, receptor, membrane transport, ion transport, channel, ATPase), and the GO *cellular component* was searched for genes related to a specific cellular structure or organelle (like, for example, extracellular matrix or mitochondrial). In a second step, differentially expressed genes between rete tissue from yellow and silver eels were identified using DESeq, the cut-off for significance was set to p < 0.01.

### Protein extraction, digestion and iTRAQ labeling

Sample preparation was performed as previously described with an additional iTRAQ labeling of peptides [35]. Briefly, frozen tissues of 12 samples (6 biological replicates for yellow and for silver eels, respectively) were homogenized and centrifuged at 1000 × g for 10 min at 4 °C to obtain a pellet containing debris and nuclear components. The supernatant was removed and centrifuged again at 15,000 × g for 30 min at 4 °C to separate the components into a membrane fraction (pellet) and cytosolic fraction (supernatant). The membrane pellets were reconstituted in 60 µl of a chaotropic buffer containing 7 M urea, 2 M thiourea, 4% CHAPS, 100 mM DTT, and 50 mM TEAB. Protein amounts were estimated using a Bradford protein assay (Carl Roth GmbH, K880). Protein samples (100 µg) were digested with Trypsin (1:50 w/w, Promega, V5117) using the FASP procedure [62].

Resulting peptides were enriched and desalted using C18 packed pipette tips (Pierce, #87,784), lyophilized, and reconstituted in 90 µl 100 mM TEAB buffer. An aliquot of 18 µl per sample was mixed with iTRAQ-4plex labeling reagent following the manufacturer’s instructions (Applied Biosystems). Samples were incubated for 3 h at room temperature, dried down in a Speed Vac and reconstituted in 75 µL 0.1% TFA. The contents of four differentially iTRAQ labeled samples were combined and separated using high pH reverse-phase spin columns (Thermo Fisher, #84,868) into eight fractions.

### LC-MS/MS analysis and database searching

Peptides from each fraction were analyzed by LC-MS within 2 h gradients using a Dionex Ultimate 3000 system (Thermo Fisher Scientific) coupled to a Q-Exactive Plus mass spectrometer (Thermo Fisher Scientific) that was set to operate in data-dependent mode. Details were as described previously [63]. Fragment spectra were matched against the draft genome sequence of the European eel [32] [protein database (34,018 sequences, https://www.ncbi.nlm.nih.gov/assembly/GCA_000695075.1] using Sequest via the Proteome Discoverer 2.2 software (Thermo Scientific, USA). The search parameters were as follows:

Enzyme specificity was set to trypsin, carbamidomethylation of cysteine was set as a fixed modification. Oxidized methionine, acetylation and/or methionine loss at the protein N-terminus, and iTRAQ 4plex label at lysine residues and at the peptide N-terminus were set as variable modifications. A maximum of two missed cleavages were allowed. The required false positive rate was set to 1% at the peptide and at the protein level, respectively, and was calculated via the Percolator node.

### iTRAQ-based protein quantitation

For quantitation of proteins the fold changes were calculated based on iTRAQ reporter ion intensities present in MS2 scans (m/z 114, 115, 116, and 117). The reporter ion intensities were extracted using the default software settings: Only peptides that were unique to a given protein or protein group were considered for quantitation. Fragment ion tolerance was set to 20 ppm for the most confident centroid peak and co-isolation threshold was set to 50%. Normalization was performed such that the total sum of the reporter ion intensities was the same for all four sample channels. To determine differences in protein expression Significance B (Maxquant/Perseus software), a P value depending on protein intensity and protein ratio was used. Settings were: Two-tailed test, FDR threshold value 0.05, Benjamini-Hochberg correction for multiple hypothesis testing was applied.

### Data analysis

The Reactome database (https://reactome.org) [61] was used for classification of detected proteins into broad pathways. The detailed pathway and biological process analysis of expressed proteins followed the procedure described for the transcriptome analysis (see above). A Spearman’s rank-order correlation was run to determine the relationship between mRNA expression and protein abundance.

## Supplementary Information

**Additional file 1.**

**Additional file 2.**

**Additional file 3.**

**Additional file 4.**

**Additional file 5.**

## Data Availability

The transcriptome datasets generated and analyzed during the current study are available in the NCBI’s Gene Expression Omnibus and are accessible through accession number GSE172092 (https://www.ncbi.nlm.nih.gov/geo/query/acc.cgi?acc=GSE172092). The proteome datasets generated and analyzed during the current study are available in the PRIDE Proteomics Identification Database and are accessible through accession number PXD025435 (https://www.ebi.ac.uk/pride/archive/projects/PXD025435).
