# Supplementary material for: Expression of transport proteins in the rete mirabile of european silver and yellow eel
Source: BMC Genomics. 2021 Dec 2;22:866. doi: 10.1186/s12864-021-08180-2 (PMC8638102; doi:10.1186/s12864-021-08180-2)
Supplement: Supplementary file 1 — Additional file 1. [file 12864_2021_8180_MOESM1_ESM.docx]

**Suppl. file 1**

Selected genes coding for proteins connected to the formation of the extracellular matrix detected in the transcriptome and/or in the proteome. For the transcriptome the base Mean relative expression value is listed for genes detected in the proteome the relative abundance is listed.

| **Name** | **Description** | **Relative expression value** | **Relative abundance** |
| --- | --- | --- | --- |
| ats15 | a disinteg and metalloprot with tb motif 15 | 70 |  |
| ats17 | a disinteg and metalloprot with tb motif 17 | 493 |  |
| ats3 | a disinteg and metalloprot with tb motif 3 | 1665 |  |
| ats7 | a disinteg and metalloprot with tb motif 7 | 52 |  |
| ats8 | a disinteg and metalloprot with tb motif 8 | 84 |  |
| ats9 | a disinteg and metalloprot with tb motif 9 | 116 |  |
| bgh3 | transf growth factor-beta-ind protein ig-h3 | 5986 | 35530711 |
| calr | calreticulin | 2232 | 94680538 |
| co1a1  co1a2  co2a1 | collagen alpha-1 chain  collagen alpha-2 chain  collagen alpha-1 chain | 215 | 1414282072  2008979894 |
| co4a1 | collagen alpha-1 chain contains | 772 | 74284731 |
| co4a2  co5a1 | collagen alpha-4 chain  collagen alpha-1 chain flags | 118 | 99759909 |
| co6a1 | collagen alpha-1 chain flags | 1259 | 346857600 |
| co6a2 | collagen alpha-2 chain flags | 3226 | 271814665 |
| co6a3 | collagen alpha-3 chain flags | 6700 | 1254133776 |
| co6a6 | collagen alpha-6 chain flags | 70 | 25160888 |
| coaa1 | collagen alpha-1 chain flags | 111 |  |
| coca1 | collagen alpha-1 chain flags | 3457 | 179391064 |
| coea1 | collagen alpha-1 chain |  | 4979255 |
| coga1 | collagen alpha-1 chain flags | 1483 |  |
| col18a1 | collagen alpha-1(XVIII) |  |  |
| cola1 | collagen alpha-1 chain flags | 74 | 407381 |
| cooa1 | collagen alpha-1 chain flag | 126 |  |
| cspg2 | versican core protein | 178 | 135853 |
| derm | dermatopontin | 724 | 3916238 |
| egfl6 | epidermal growth factor-like protein 6 | 209 |  |
| emil1 | emilin-1 | 11332 | 195152499 |
| fbln1 | fibulin-1 | 550 | 19606196 |
| fbln3 | egf-cont fibulin-like extrac matrix prot 1 | 178 | 244280 |
| fbln5 | fibulin-5 | 278 | 479153 |
| fgfr2 | fibroblast growth factor receptor 2 | 645 |  |
| finc | fibronectin | 1232 | 451690479 |
| gdn | glia-derived nexin | 166 | 727268 |
| gpc4 | glypican-4 | 4414 | 18763947 |
| hmcn1 | hemicentin-1 | 128 | 781011151 |
| l2gl2 | lethal giant larvae protein homolog 2 | 431 | 2451633 |
| lama2 | laminin subunit alpha-2 | 116 |  |
| lama3 | laminin subunit alpha-3 | 4814 | 57800518 |
| lama5 | laminin subunit alpha-5 | 224 | 63714252 |
| lamc1 | laminin subunit gamma-1 | 56 | 320195649 |
| lamc2 | laminin subunit gamma-2 | 86 |  |
| lamc3 | laminin subunit gamma-3 | 342 |  |
| leg3 | galectin-3 | 890 | 19737545 |
| megf6 | multiple egf-like domains protein 6 | 1694 | 11254611 |
| mfap2 | microfibrillar-associated protein 2 | 215 | 14672111 |
| mime | mimecan | 99 | 17591363 |
| mmp13  mmp14 | matrix metalloproteinase-13  matrix metalloproteinase-14 | 2587 | 63714252 |
| mmp19 | matrix metalloproteinase-19 | 467 |  |
| mmp2 | 72 kda type iv collagenase | 2464 |  |
| mmp23 | matrix metalloproteinase-23 | 65 |  |
| mmp24 | matrix metalloproteinase-24 | 69 |  |
| mmp28 | matrix metalloproteinase-28 | 67 |  |
| mrc2 | c-type mannose receptor 2 | 252 | 21989153 |
| nid1 | nidogen-1 | 1076 | 103044500 |
| nid2 | nidogen-2 | 13634 | 852851 |
| pgbm | base memb-spec heparan sulfate proteoglyc core protein | 2426 | 527875835 |
| postn | periostin | 381 | 1504036 |
| ppn | papilin flags | 2109 | 239312 |
| ptprb | rec-type tyrosine-protein phosphatase beta | 880 | 31636161 |
| rell1 | relt-like protein 1 | 168 | 2322177 |
| se1l1 | protein sel-1 homolog 1 | 101 | 15175741 |
| spon1 | spondin-1 | 891 | 1636746 |
| stx2 | syntaxin-2 | 174 | 36801411 |
| stx4 | syntaxin-4 | 252 | 30929702 |
| tgbr3 | transf growth factor beta receptor type 3 | 4780 | 5377726 |
| thsd4 | thrombosp type-1 domain-cont protein 4 | 73 | 22958706 |
| wnt11 | protein wnt-11 | 3129 | 2016317 |
