# Supplementary material for: Expression of transport proteins in the rete mirabile of european silver and yellow eel
Source: BMC Genomics. 2021 Dec 2;22:866. doi: 10.1186/s12864-021-08180-2 (PMC8638102; doi:10.1186/s12864-021-08180-2)
Supplement: Supplementary file 2 — Additional file 2. [file 12864_2021_8180_MOESM2_ESM.docx]

**Suppl. file 2**

Selected genes coding for membrane transport proteins detected in the transcriptome and/or in the proteome. For the transcriptome the base Mean relative expression value is listed, for genes detected in the proteome the relative abundance is listed.

| **Name** | **Description** | **Relative expression value** | **Relative abundance** |
| --- | --- | --- | --- |
| aqp1 | aquaporin-1 | 1627 | 11660053 |
| at1a1 | sodium potassium-transp atpase subunit alpha-1 | 4707 | 1007642829 |
| at1a3 | sodium potassium-transp atpase subunit alpha-3 | 1634 | 6242077 |
| at1b1 | sodium potassium-transp atpase subunit beta-1 | 1385 | 104122831 |
| at1b3 | sodium potassium-transp atpase subunit beta-3 | 147 | 12196693 |
| at233 | sodium potassium-transp atpase subunit beta-233 | 757 | 22380290 |
| at2a1 | sarcoplasmic endopl reticulum calcium atpase 1 | 760 | 171278881 |
| at2a2 | sarcoplasmic endopl reticulum calcium atpase 2 | 2061 | 352265687 |
| at2b2 | plasma membrane calcium-transporting atpase 2 |  | 285083842 |
|  |  |  |  |
| at2b4 | plasma membrane calcium-transporting atpase 4 | 624 | 6910686 |
| at5f1 | atp synthase subunit mitochondrial | 218 | 209849099 |
|  |  | |  |
| ca2d1 | voltage-dep calcium channel subunit alpha-2 delta-1 | 314 | 6413556 |
| ca2d2 | voltage-dep calcium channel subunit alpha-2 delta-2 | 351 | 1736966 |
| ca2d4 | voltage-dep calcium channel subunit alpha-2 delta-4 | 123 |  |
| cac1c | voltage-dep l-type calcium channel subunit alpha-1c | 96 |  |
|  |  | |  |
| cac1f | voltage-dep l-type calcium channel subunit alpha-1f | 105 |  |
| cac1g | voltage-dep t-type calcium channel subunit alpha-1g | 54 |  |
| cac1h | voltage-dep t-type calcium channel subunit alpha-1h | 498 | 2153070 |
| cacb1 | voltage-dep l-type calcium channel subunit beta-1 | 67 |  |
| cacb3 | voltage-dep l-type calcium channel subunit beta-3 | 266 |  |
| crcm1 | calcium release-activated calcium channel protein 1 | 618 | 2367947 |
| gtr1 | solute carrier fam facilit glucose transp member 1 | 3032 | 19286778 |
| gtr10 | solute carrier fam facilit glucose transp member 10 | 80 |  |
| gtr3 | solute carrier fam facilit glucose transp member 3 | 317 | 24096696 |
| gtr6 | solute carrier fam facilit glucose transp member 6 | 100 |  |
| mot1 | monocarboxylate transporter 1 | 3725 | 1486384831 |
| mot10 | monocarboxylate transporter 10 | 229 | 5142274 |
| mot4 | monocarboxylate transporter 4 | 760 | 4980675 |
| mot6 | monocarboxylate transporter 6 | 525 |  |
| mot7 | monocarboxylate transporter 7 | 431 |  |
| mot9 | monocarboxylate transporter 9 | 181 |  |
| nhrf1 | na(+) h(+) exchange regulatory cofactor nhe-rf1 | 3934 | 141306557 |
| nkai1 | sod pot-transp atpase subunit beta-1-interact prot 1 | 82 |  |
|  |  | |  |
| s4a11 | sodium bicarbonate transporter-like protein 11 | 175 |  |
| s4a4 | electrogenic sodium bicarbonate cotransporter 1 | 202 |  |
| s4a7 | sodium bicarbonate cotransporter 3 | 11625 | 47171849 |
| so2a1 | solute carrier organic anion transp fam member 2a1 | 1726 | 29522545 |
| so2b1 | solute carrier organic anion transp fam member 2b1 | 202 |  |
| so3a1 | solute carrier organic anion transp fam member 3a1 | 559 |  |
| so5a1 | solute carrier organic anion transp fam member 5a1 | 410 |  |
| va0d1 | v-type proton atpase subunit d 1 | 1567 | 18210116 |
| va0e2 | v-type proton atpase subunit e 2 | 161 |  |
| vas1 | v-type proton atpase subunit s1 | 452 |  |
| vata | v-type proton atpase catalytic subunit a | 969 | 35937475 |
| vatb | v-type proton atpase subunit b | 140 |  |
| vatd | v-type proton atpase subunit d | 345 | 2842915 |
| vate1 | v-type proton atpase subunit e 1 | 426 | 10365811 |
| vatf | v-type proton atpase subunit f | 207 | 294973 |
| vatg1 | v-type proton atpase subunit g 1 1 | 1071 | 8298594 |
| vath | v-type proton atpase subunit h | 741 | 1818928 |
| vatl | v-type proton atpase 16 kda proteolipid subunit | 318 |  |
| vatm | vacuolar proton translocating atpase 100 kda subunit | 123 |  |
| vato | v-type proton atpase 21 kda proteolipid subunit | 796 |  |
| vma21 | vacuolar atpase assembly integral membrane protein | 643 | 2441689 |
| vpp1 | v-type proton atpase 116 kda subunit a isoform 1 | 3925 | 15461334 |
| vpp2 | v-type proton atpase 116 kda subunit a isoform 2 | 327 | 1015813 |
| vpp3 | v-type proton atpase 116 kda subunit a isoform 3 | 276 | 2800503 |
| vps4a | vacuolar protein sorting-associated protein 4a | 452 | 1061772 |
| vps4b | vacuolar protein sorting-associated protein 4b | 2283 |  |
| vtc1a | v-type proton atpase subunit c 1-a | 705 | 2425462 |
|  |  |  |  |
