# Supplementary material for: Expression of transport proteins in the rete mirabile of european silver and yellow eel
Source: BMC Genomics. 2021 Dec 2;22:866. doi: 10.1186/s12864-021-08180-2 (PMC8638102; doi:10.1186/s12864-021-08180-2)
Supplement: Supplementary file 3 — Additional file 3. [file 12864_2021_8180_MOESM3_ESM.docx]

**Suppl. file 3**

Selected genes coding for receptor proteins detected in the transcriptome and/or in the proteome. For the transcriptome the base Mean relative expression value is listed, for genes detected in the proteome the relative abundance is listed.

| **Name** | **Description** | **Relative expression value** | **Relative abundance** |
| --- | --- | --- | --- |
| 5ht3a | 5-hydroxytryptamine receptor 3a | 1463 |  |
| aa2ar | adenosine receptor a2a | 376 |  |
| aa2db | alpha-2db adrenergic receptor | 182 |  |
| acha | acetylcholine receptor subunit alpha | 1027 |  |
| acha4 | neuronal ach receptor subunit alpha-4 | 348 |  |
| acha7 | neuronal ach receptor subunit alpha-7 | 302 |  |
| ache | acetylcholine receptor subunit epsilon | 72 |  |
| acm2 | muscarinic acetylcholine receptor m2 | 65 |  |
| ada1b | alpha-1b adrenergic receptor | 73 |  |
| ada1d | alpha-1d adrenergic receptor | 93 |  |
| ada2a | alpha-2a adrenergic receptor | 521 |  |
| ada2b | alpha-2b adrenergic receptor | 54 |  |
| agtr2 | type-2 angiotensin ii receptor | 334 |  |
| agtrb | type-1 angiotensin ii receptor b | 908 |  |
| ahr | aryl hydrocarbon receptor | 6007 | 290516 |
| anpra | atrial natriuretic peptide receptor 1 | 585 | 2813878 |
| anprb | atrial natriuretic peptide receptor 2 | 390 |  |
| anprc | atrial natriuretic peptide receptor 3 | 469 |  |
| arbk2 | beta-adrenergic receptor kinase 2 |  | 1419752 |
| arnt | aryl hydrocarb rec nuclear translocator | 221 |  |
| arnt2 | Arnt 2 | 1263 |  |
| bmal1 | arnt-like protein 1 | 68 |  |
| bmal2 | arnt-like protein 2 | 527 |  |
| calcr | calcitonin receptor | 1904 | 955035 |
| calrl | calcitonin gene-related peptide type 1 rec | 12126 | 22082123 |
| celr1 | cadherin egf lag seven-pass g-type rec 1 | 116 |  |
| ednra | endothelin-1 receptor | 13414 | 20738070 |
| ednrb | endothelin b receptor | 2075 | 1386728 |
| egfr | epidermal growth factor receptor | 972 |  |
| emr1 | egf-like module-containing mucin-like hormone receptor-like 1 | 81 |  |
| ep15r | egf receptor substrate 15-like 1 | 1423 |  |
| eps15 | egf receptor substrate 15 | 98 | 19564489 |
| eps8 | egf receptor kinase substrate 8 | 653 |  |
| es8l3 | egf rec kinase substrate 8-like prot 3 | 726 |  |
| fgfr2 | fibroblast growth factor receptor 2 | 645 |  |
| fgr1a | basic fibroblast growth factor receptor 1-a | 470 |  |
| gcr | glucocorticoid receptor | 697 | 714308 |
| gria3 | glutamate receptor 3 | 61 |  |
| gria4 | glutamate receptor 4 | 120 |  |
| grlf1 | glucocorticoid receptor dna-binding factor 1 | 774 | 8700333 |
| igf1r | insulin-like growth factor 1 receptor | 88 | 2150126 |
| il1ap | interleukin-1 receptor accessory protein | 328 | 2526737 |
| ingr1 | interferon gamma receptor 1 | 2651 | 1714075 |
| insr | insulin receptor | 1036 | 21630632 |
| irak1 | interleukin-1 receptor-associated kinase 1 | 179 |  |
| irak3 | interleukin-1 receptor-associated kinase 3 | 427 | 259456 |
| itpr1 | inositol -trisphosphate receptor type 1 | 1180 | 9282077 |
| itpr2 | inositol -trisphosphate receptor type 2 |  | 1906923 |
| itpr3 | inositol -trisphosphate receptor type 3 | 167 |  |
| met | hepatocyte growth factor receptor | 2086 | 14031732 |
| pr285 | ppar-activated receptor a-interacting complex 285 kda protein | 1703 | 256182 |
| ptpre | recr-type tyrosine-protein phosph epsilon | 1421 | 1929484 |
| ptprg | rec-type tyrosine-protein phosph gamma | 20799 | 1353566177 |
| tgbr3 | transf growth factor beta receptor type 3 | 4780 | 5377726 |
| tgfa1 | tgf-beta receptor-associated prot 1 homolog | 2094 | 8333133 |
| tgfr2 | tgf-beta receptor type-2 | 11204 | 7793635 |
| tyro3 | tyrosine-protein kinase receptor tyro3 | 322 | 2242395 |
| tie1 | tyrosine-protein kinase receptor tie-1 | 2234 | 4700628 |
| tie2 | tyrosine-protein kinase receptor tie-2 | 8800 | 12840248 |
| vgfr2 | vegf receptor 2 | 6609 | 1846386 |
| vgfr3 | vegf 3 | 1552 |  |
| vgfr4 | vegf kdr-like | 3837 | 15607904 |
|  |  |  |  |
