# Supplementary material for: Expression of transport proteins in the rete mirabile of european silver and yellow eel
Source: BMC Genomics. 2021 Dec 2;22:866. doi: 10.1186/s12864-021-08180-2 (PMC8638102; doi:10.1186/s12864-021-08180-2)
Supplement: Supplementary file 5 — Additional file 5. [file 12864_2021_8180_MOESM5_ESM.docx]

**Suppl. file 5**

Between yellow and silver eels differentially expressed genes detected in the proteome (calculated in Perseus).

| **Name** | **Description** | **FoldChange (yellow/silver)** | **Relative  Abundance** |
| --- | --- | --- | --- |
| a2mg | alpha-2-macroglobulin-like isoform X1 | 2,110 | 415716 |
| anxa4 | annexin A4-like | 0,587 | 615240532 |
| at1b4 | protein ATP1B4 | 2,331 | 476901 |
| at1b4 | protein ATP1B4-like | 2,197 | 932547 |
| b2mg | beta-2-microglobulin | 1,541 | 55135850 |
| bgh3 | transf. growth factor-beta-induced prot. | 1,420 | 35530711 |
| cd052 | small integral membrane protein 20 | 1,804 |  |
| clus | clusterin | 2,292 | 279535 |
| co6a1 | collagen alpha-1(VI) chain-like | 2,040 | 298012183 |
| co6a2 | collagen alpha-2(VI) chain-like isof. X1 | 2,319 | 132058224 |
| co6a2 | collagen alpha-2(VI) chain-like isof. X1 | 1,950 | 139756442 |
| co6a3 | collagen alpha-3(VI) chain-like isof. X1 | 2,047 | 742512966 |
| co6a6 | collagen alpha-6(VI) chain-like isof. X1 | 1,827 | 25160888 |
| derm | dermatopontin | 1,725 | 3916238 |
| frpa | uncharact. prot. LOC118219097 isof. | 2,045 | 1680042 |
| gfap | glial fibrillary acidic protein | 1,444 | 23759027 |
| glpk | glycerol kinase-like | 2,542 | 416524 |
| gvin1 | interf.-induced very large GTPase 1-like | 1,547 | 2696312 |
| h2a | late histone H2A.2.2-like | 1,611 | 1449521458 |
| h2av | histone H2A.V | 1,498 | 286011409 |
| h2ax | histone H2AX | 2,545 | 5978615 |
| h2b | histone H2B-like | 1,501 | 2991408282 |
| hbaa | hemoglobin anodic subunit alpha | 1,434 | 293766635 |
| hbba | ba1 globin, like | 1,891 | 601913638 |
| hcfc1 | host cell factor 1b isoform X3 | 2,111 | 445245 |
| hecd3 | E3 ubiquitin-protein ligase HECTD3 | 2,115 |  |
| il1ap | interleukin-1 receptor acc.y prot. isof. X1 | 0,413 | 2526737 |
| ion3 | intermediate filament protein ON3-like | 1,483 | 3177198 |
| l3bpb | galectin-3-binding protein A-like | 2,550 | 274507 |
| lpp3 | phospholipid phosphatase 3 isoform X2 | 2,343 | 3528592 |
| lrc15 | leucine-rich repeat-cont. protein 15-like | 2,966 | 927012 |
| marcs | myristoyl. ala.-rich prot. kinase C subst. | 0,521 | 39259783 |
| mime | osteoglycin, paralog b | 1,805 | 17591363 |
| myl6 | myosin light polypeptide 6 isoform X4 | 1,750 | 8344842 |
| myl9 | myosin regulatory light polypeptide 9 | 1,528 | 96557006 |
| nibl1 | protein Niban 2a | 1,573 | 6227327 |
| nid1 | nidogen-2 isoform X2 | 1,472 | 71677445 |
| pi3r4 | phosphoinositide 3-kinase regulat. subunit 4 isof. | 1,826 | 349878 |
| pnmt | phenylethanolamine N-methyltransferase | 1,562 | 1968289 |
| postn | periostin, osteoblast specific factor b isoform X1 | 1,723 | 1504036 |
| rpesp | somatomedin-B and thrombospondin type-1  domain-containing protein | 2,284 | 671054 |
| rs28 | 40S ribosomal protein S28-like | 0,520 | 31757135 |
| rtn4 | reticulon-1-A-like isoform X6 | 1,765 | 1365034 |
| scrb2 | lysosome memb. prot. 2-like isoform X1 | 0,518 | 68263076 |
| tinal | tubulointerstit. nephritis antigen-like isof. X1 | 1,700 | 58817815 |
| tsn8 | tetraspanin-8-like | 1,834 |  |
| ubp2l | ubiquitin-associated prot. 2-like isof. X1 | 2,183 | 560875 |
| vrk1 | serine/threonine-protein kinase VRK1 | 1,512 | 5386068 |
| wahs7 | WASH complex subunit 4 | 2,492 |  |
| - | asialoglycoprotein receptor 1-like | 1,675 | 24047 |
| - | carbonic anhydrase 14 | 2,953 |  |
| - | chromobox protein homolog 3a | 1,663 | 1646404 |
| - | class I histocompat. antigen, F10 alpha chain-like | 1,646 |  |
| - | core histone macro-H2A.1 isoform X1 | 1,469 | 17388220 |
| - | dual specificity protein phosphate 18-like | 2,592 |  |
| - | fucolectin-7-like isoform X2 | 3,681 |  |
| - | galactose-binding lectin l-1-like | 1,846 | 24946 |
| - | galactose-binding lectin l-1-like | 1,544 |  |
| - | GTPase IMAP family member 8-like isoform X1 | 2,215 |  |
| - | guanylate-binding protein 2-like | 2,039 | 1671230 |
| - | hemoglobin cathodic subunit alpha | 1,834 | 225946017 |
| - | hemoglobin cathodic subunit beta | 1,814 | 441801363 |
| - | hemopexin-like | 1,530 | 134946850 |
| - | interferon-induced GTP-bind. prot. Mx3-like | 1,505 | 6033833 |
| - | mucin-5AC-like isoform X2 | 0,051 |  |
| - | Na(+)/H(+) exchange regulatory cofactor NHE-RF2 | 2,042 | 673078 |
| - | NADPH:adrenodoxin oxidoreductase, mitochondrial  isoform X1 | 1,791 | 4266582 |
| - | NF-kappa-B essential modul. Isof. | 2,819 | 688625 |
| - | platelet endothelial cell adhesion molec. isof. X6 | 0,530 | 49271181 |
| - | retinal rod rhodopsin-sensitive cGMP 3',5'-cyclic  phosphodiesterase subunit delta | 2,606 |  |
| - | serglycin-like | 2,309 |  |
| - | synembryn-A | 3,751 | 110023 |
| - | teneurin-1 isoform X1 | 2,470 |  |
| - | visinin-like protein 1a | 2,248 | 275691 |
